# Structural insights into ligand recognition and activation of human purinergic receptor P2Y14

**DOI:** 10.1038/s41421-025-00799-9

**Published:** 2025-05-13

**Authors:** Quanchang Gu, Zhenyu Lv, Tianxin Wang, Wenqin Tang, Xuzhen Guo, Xiangling Huang, Fahui Li, Jiangyun Wang

**Affiliations:** 1 CAS Key Laboratory of Quantitative Engineering Biology, Institute of Synthetic Biology, Shenzhen Institute of Advanced Technology, Chinese Academy of Sciences, Shenzhen, Guangdong China; 2 Key Laboratory of Biomacromolecules (CAS), CAS Center for Excellence in Biomacromolecules, Institute of Biophysics, Chinese Academy of Sciences, Beijing, China; 3 School of Life Sciences, University of Chinese Academy of Sciences, Beijing, China; 4 iHuman Institute, ShanghaiTech University, Shanghai, China; 5 School of Life Science and Technology, ShanghaiTech University, Shanghai, China

**Keywords:** Extracellular signalling molecules, Cryoelectron microscopy

Dear Editor,

Purinergic receptors, including P2Y1-like receptors (P2Y1, 2, 4, 6, and 11) that signal through G_q/11_ proteins and P2Y12-like receptors (P2Y12, 13, and 14) that activate G_i/o_ proteins, are involved in diverse physiological processes such as cell proliferation, chemotaxis, inflammation, cancer metastasis, cardiovascular events, neurodegenerative diseases, and aging. To date, active and inactive structures of P2Y1 and P2Y12 have been resolved, providing structural insights into P2Y receptor signaling mechanisms^1^. UDP-sugars, including UDP-glucose (UDPG), UDP-glucuronic acid (UDPGA), UDP-galactose, and UDP-*N*-acetylglucosamine, are produced in the cytoplasmic matrix under normal physiological conditions and play important roles in regulating blood sugar levels, fat metabolism, and inflammation^2,3^. UDP-sugars are secreted into the extracellular space to function as the paracrine signals through the G-protein-coupled purinergic receptor, P2Y14, which senses extracellular metabolic stress signals and regulates energy homeostasis^4,5^. P2Y14 couples to G_i_ signaling pathways, orchestrating a wide range of biological processes ranging from gastric function to immune responses, renal inflammation, and liver fibrosis^6–8^. Therefore, P2Y14 has emerged as a promising therapeutic target for asthma, acute kidney injury, and inflammatory bowel disease (IBD) (Fig. 1a)^9–11^. Despite its significance, the molecular activation mechanism underlying its ability to recognize diverse ligands and couple G_i_ protein remains elusive. There is an urgent need to delineate the molecular mechanism underlying P2Y14 signaling.

**Fig. 1:**
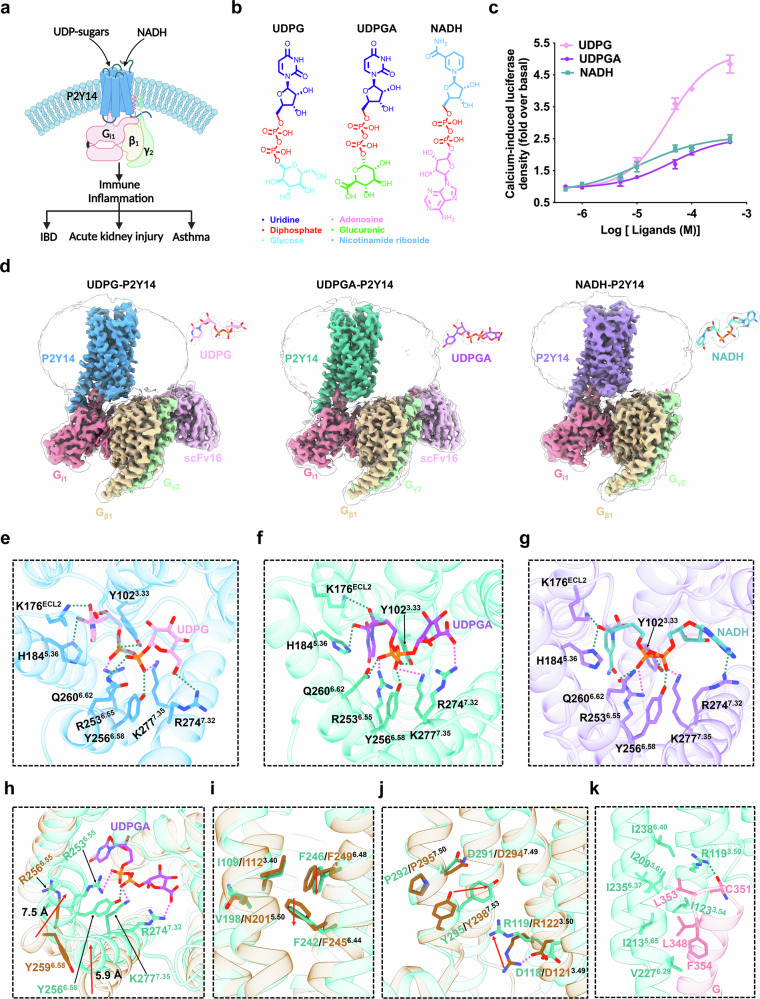
Structure basis of P2Y14 activation. **a** Schematic representation of P2Y14-mediated activation of downstream G_i_ protein signaling pathways in response to various ligands. **b** Chemical structures of the endogenous agonists used in this study. The chemical groups of endogenous agonists were represented in distinct colors. **c** Agonist potency on G-protein signaling in P2Y14, analyzed using a calcium-induced luciferase accumulation assay. Data are shown as the mean ± SEM. from three independent measurements. **d** Cryo-EM density maps of the UDPG-, UDPGA-, and NADH-bound P2Y14–G_i_ complexes. **e**–**g** Interactions between UDPG (pink) with P2Y14 (blue) (**e**), UDPGA (violet) with P2Y14 (strong cyan) (**f**), and NADH (cyan) with P2Y14 (purple) (**g**). **h** Structural comparison between the active P2Y14 (strong cyan) and inactive P2Y12 (dark orange). **i** Structural rearrangement of the PIF triad and toggle switch during P2Y14 activation. **j** Structural rearrangement of the E/DRY and NPXXY motifs during P2Y14 activation. **k** Detailed interactions of P2Y14 with the α5-helix of G_i_ in the UDPGA-bound P2Y14 structure. Hydrogen-bonding interactions are shown with green dashed lines, and ionic interactions are shown with pink dashed lines.

Endogenous UDP-sugars share common moieties, such as uridine and diphosphate groups, with their structural variations primarily arising from differences in the attached sugar groups. By comparing the structures of UDP-sugars with endogenous metabolites, we found that NADH exhibits characteristic chemical scaffolds, such as diphosphate and nicotinamide riboside, which are structurally similar to the uridine and diphosphate groups found in UDP sugars (Fig. 1b), suggesting that NADH may function as an endogenous agonist of P2Y14. To test our hypothesis, calcium-induced luciferase accumulation assays were performed to measure G_q/i_-protein subtype activation in the presence of NADH. The results of the cellular assays showed that P2Y14 was activated by NADH in a dose-dependent manner, confirming that NADH functions as an endogenous agonist of P2Y14 (Fig. 1c).

To investigate the molecular activation and G_i_ coupling mechanisms of P2Y14, we determined the cryo-electron microscopy (cryo-EM) structures of the G_i_ heterotrimer-coupled P2Y14 in complex with UDPG, UDPGA, and NADH, respectively. To obtain stable and homogeneous P2Y14–G_i_ complexes, a BRIL tag was fused to the N-terminus of P2Y14, and a dominant-negative G_i_ was employed to facilitate complex assembly. The active-state structures of P2Y14 bound to the G_i_ heterotrimer and the endogenous agonists were further stabilized using a single-chain antibody scFv16 (Supplementary Figs. S1a, b, S2a, b, S3a, b). Unexpectedly, the electron density for scFv16 was not observed in the map of the NADH-bound P2Y14–G_i_ complex (Fig. 1d). The global resolutions of the G_i_-coupled P2Y14 structures with UDPG, UDPGA, and NADH were determined to be 2.88 Å, 2.76 Å, and 2.76 Å, respectively (Supplementary Figs. S1c–h, S2c–h, S3c–h and Table S1). These structures exhibit a similar folding configuration, with the root mean square deviation (RMSD) values being less than 0.5 Å between the complexes (Supplementary Fig. S4a). The relatively high-quality density maps allowed accurate modeling of the seven-transmembrane (7TM) cores of the receptors, the G_i_ heterotrimer, and scFv16. In addition, the side chains of most residues are well-defined in all components, providing a precise modeling of the intermolecular interactions of P2Y14 with the agonists and G_i_ protein (Fig. 1d; Supplementary Figs. S1i, S2i, S3i).

Despite variations in agonist structures, the active-state orthosteric pockets in UDPG-, UDPGA-, and NADH-bound P2Y14 complexes share common features, consisting of residues in transmembrane helices TM2–TM7. The extracellular loop 2 (ECL2) acts as a “lip”, anchoring the agonists within the orthosteric pocket (Supplementary Fig. S4b). These agonists occupy an upper binding site in the receptor’s extracellular region, with the uridine groups in UDPG/UDPGA and the nicotinamide group in NADH penetrating deeply into a narrow cavity within the transmembrane core, where they form extensive interactions with TM4 and TM5. In contrast, the sugar groups of UDPG/UDPGA and the adenosine group of NADH face the extracellular surface, interacting with TM2 and TM7. Structural comparisons with other purinergic receptors, such as P2Y1 and P2Y12, reveal that the binding pockets of P2Y14 and P2Y12 extend deeper into the extracellular cavity than that of P2Y1 (Supplementary Fig. S4c). The NADH-binding pocket in P2Y14 is larger than those for UDPG and UDPGA, with volumes ranging from 443 Å³ to 512 Å³, suggesting that the distinct geometry of NADH results in a larger binding pocket (Supplementary Fig. S4c). Due to the structural similarities among the agonists, the UDPG-, UDPGA-, and NADH-bound P2Y14 complexes share common ligand-interacting residues at the extracellular vestibule. These agonists primarily interact with the orthosteric pocket of P2Y14 through hydrogen-bonding and ionic interactions. The uridine groups of UDPG/UDPGA and the nicotinamide riboside group of NADH form hydrogen-bonding interactions with residues K176^ECL2^ and H184^5.36^ (superscripts indicate Ballesteros-Weinstein numbering for G-protein-coupled receptors (GPCRs)), along with π–π stacking interactions with Y102^3.33^ (Fig. 1e–g; Supplementary Fig. S5a–c). The diphosphate groups of these agonists are anchored through ionic interactions with residues R253^6.55^ and K277^7.35^, as well as hydrogen-bonding interactions with Y102^3.33^, Y256^6.58^, and Q260^6.62^ (Fig. 1e–g; Supplementary Fig. S5a–c). Additionally, the residue R274^7.32^ forms hydrogen-bonding and ionic interactions with the sugar groups of UDPG/UDPGA and the adenosine group of NADH (Fig. 1e–g; Supplementary Fig. S5a–c). In functional assays, mutations of these interacting residues resulted in a decreased agonist potency for UDPG, UDPGA, and NADH (Supplementary Fig. S5d–h and Table S2). Beyond these common interactions, other residues in P2Y14 engage in specific polar and hydrophobic interactions with the agonists, creating distinct interaction patterns. For example, UDPG interacts with D81^2.64^ and N156^4.60^; UDPGA with K77^2.60^, N90^3.21^, N156^4.60^, and N188^5.40^; and NADH with K77^2.60^, Y106^3.37^, N188^5.40^, F191^5.43^, and E278^7.36^ (Supplementary Fig. S5a–c, i). The subtle structural differences between UDPG and UDPGA arise from their sugar groups, which contribute to distinct agonist recognition patterns. The residues D81^2.64^ and N90^3.21^ establish hydrogen-bonding interactions with UDPG and UDPGA, respectively. In functional assays, the D81^2.64^A mutation led to a more substantial decrease in UDPG activity compared to that in UDPGA. Conversely, the N90^3.21^A mutation caused a more significant reduction in UDPGA activity than in UDPG (Supplementary Fig. S5a, b, g). Additionally, NADH exhibits unique ligand–receptor interactions by forming hydrogen bonds with residues Y106^3.37^ and E278^7.36^. Y106^3.37^A and E278^7.36^A mutants of P2Y14 showed significantly reduced activation by NADH relative to UDPG and UDPGA (Supplementary Fig. S5a–g). Notably, previous study reported that K77^2.60^ is crucial for the recognition of the sugar groups in UDP-sugars^12^. However, in the UDPG-bound P2Y14 structure, K77^2.60^ is distant from the UDP-sugars and instead forms a salt bridge with E278^7.36^, stabilizing the architecture of the orthosteric cavity (Supplementary Fig. S6a). The mutation at K77^2.60^ resulted in a larger decrease in the activity of UDPG compared to the mutation at E278^7.36^, which can be attributed to the significantly higher structural instability observed in the K77^2.60^A mutant (Supplementary Fig. S6b, c). To further validate the agonist-binding poses, we performed all-atom molecular dynamics (MD) simulations (400 ns) on these P2Y14 systems. RMSD results showed that UDPG, UDPGA, and NADH remained close to their initially modeled poses throughout the simulations (Supplementary Fig. S7).

P2Y14 belongs to the δ-branch GPCR subfamily, including SUCR1, P2Y1, P2Y12, HCA2, CysLT1/2, PAR1/2, PAFR, GPR35, and LPA6. The lack of an inactive P2Y14 structure has hindered the understanding of its activation mechanism. The closest phylogenetic neighbor of P2Y14 is P2Y12 receptor that also couples to G_i_ protein. Therefore, the determined structure of the AZD1283-bound inactive P2Y12 complex (PDB: 4NTJ) allowed us to infer the activation mechanism of P2Y14. A comparison of the UDPGA-bound active P2Y14 with the AZD1283-bound inactive P2Y12 revealed distinct conformations on both the extracellular and intracellular sides. In the P2Y14 structure, UDPGA binding causes inward movements of the extracellular region of TM6 for ~7.5 Å (relative to the α-carbon distance between K258^6.60^ in P2Y14 and L261^6.60^ in P2Y12) and that of TM7 for ~5.9 Å (relative to the α-carbon distance between S266^7.24^ in P2Y14 and D269^7.24^ in P2Y12), along with an outward shift in TM6’s intracellular portion for ~7.8 Å (relative to the α-carbon distance between K228^6.30^ in P2Y14 and R231^6.30^ in P2Y12) compared to P2Y12 (Fig. 1h; Supplementary Fig. S8a, b). The agonist-induced upward shift of TM3 is a key activation feature in δ-branch receptors like P2Y1, P2Y12, HCA2, and SUCR1^13^, driven by interactions with a positively charged residue in TM3. However, this shift is absent in active P2Y14, suggesting that P2Y14 activation is independent of TM3’s conformational rearrangement. Additionally, P2Y14 lacks the characteristic proline kink (P^5.50^) found in most class A GPCRs, where it typically induces a bend in TM5 during transducer coupling. Instead, P2Y14 has V198^5.50^, which results in a straight TM5, similar to P2Y12 and HCA2 (Supplementary Fig. S8c–e). Moreover, the conserved toggle switch residue in the majority of class A GPCR is W^6.48^. However, the toggle switch at position 6.48 is occupied by a less bulky residue (F/Y/L^6.48^) in δ-branch GPCRs, leading to a toggle switch movement rather than the typical W^6.48^ rotation during activation. In the inactive P2Y12 structure, the side chains of the residues R256^6.55^ and Y259^6.58^ in TM6 are positioned away from the orthosteric pocket (Fig. 1h). Upon UDPGA binding to P2Y14, the side chains of R253^6.55^ and Y256^6.58^ rotate towards the orthosteric pocket, forming polar interactions with UDPGA and triggering downward shifts of toggle switch F246^6.48^ and F242^6.44^ of the PIF triad (V198^5.50^I109^3.40^F242^6.44^) (Fig. 1i). Additionally, UDPGA binding also establishes hydrogen-bonding interactions with the residues R274^7.32^ and K277^7.35^ in TM7, inducing inward movement of TM7’s extracellular region. Subsequently, conformational rearrangements of the conversed E/DR^3.50^Y and N^7.49^P^7.50^XXY^7.53^ motifs (DR119^3.50^Y and D291^7.49^PVFY in P2Y14) are triggered simultaneously. In the UDPGA-bound P2Y14 structure, the side chain of R119^3.50^ extends toward TM7 and packs closely with the D291^7.49^PVFY motif compared to the inactive P2Y12 structure (Fig. 1i, j), allowing for the insertion of the C-terminus of the G_i_ protein. Together, these findings indicate that P2Y14 activation involves a cascade of conformational changes, particularly in TM6 and TM7, transmitting the activation signal from the extracellular region to the intracellular portion.

The structural determination of the P2Y14–G_i_ complexes offers valuable insights for dissecting the G_i_ coupling mechanism. Given the higher resolution of the UDPGA-bound P2Y14 structure, it was selected for detailed analysis of the receptor–G_i_ protein interactions. Structural comparisons of UDPGA-bound P2Y14–G_i_ with other G_i_-coupled GPCR structures revealed that the α5-helix of G_i_ in P2Y14–G_i_ closely resembles that of GPR84–G_i_ in terms of orientation and movements (Supplementary Fig. S9). In the active P2Y14 complex, the interactions between G_i_ and the cytoplasmic cavity of P2Y14 are primarily contributed by TM3, TM5, and TM6 of the receptors (Fig. 1k). The α5 helix of G_i_ is amphipathic and forms extensive hydrophobic and hydrogen-bonding interactions with the cytoplasmic cavity of P2Y14. The residue R119^3.50^ of the DRY motif establishes hydrogen-bonding interaction with the α5-helix backbone of G_i_ protein, playing a key role in G-protein activation. Mutation of this residue in P2Y14 reduced G_i_ protein signaling (Supplementary Fig. S10a–c). Additionally, the side chains of F354, F353, C351, and L348 in the α5-helix of G_i_ are involved in extensive hydrophobic interactions with the hydrophobic pocket (I123^3.54^, I209^5.61^, I213^5.65^, V227^6.29^, I235^6.37^, and I238^6.40^) in P2Y14 (Fig. 1k). These hydrophobic interactions of P2Y14 with G_i_’s α5-helix were further confirmed by the functional assays (Supplementary Fig. S10a–c).

P2Y14 is widely distributed in placenta, spleen, bone marrow, thymus, stomach, intestine, adipose tissue, lung, and brain, and has recently attracted attention as a potential drug target for asthma, acute kidney injury, and IBD. The determination of experimental P2Y14 structure is critical for understanding its pathophysiological roles and facilitating drug design. The cryo-EM structures obtained in this study elucidate the agonist recognition mechanism of P2Y14, which primarily depends on polar interactions between the agonist and the receptor. These distinct polar interaction networks within P2Y14’s orthosteric pocket offer new avenues for designing synthetic ligands with diverse structures. While agonist-induced rearrangement of TM3 is a common activation mechanism among δ-branch GPCRs, P2Y14 exhibits an atypical activation mode, driven by agonist-induced conformational changes in TM6 and TM7. NADH, a key factor in transferring electrons from the tricarboxylic acid cycle to the electron transport chain, plays a central role in energy metabolism and ATP production^14^. While earlier studies have primarily focused on NADH’s intracellular functions, recent research suggests that NADH may also act as a paracrine signal^15^. However, the molecular recognition mechanism by which extracellular NADH mediates signal transduction through membrane receptors remains unclear. Our study is the first to identify NADH as an endogenous agonist of P2Y14, supported by cellular functional assays and the active cryo-EM structure of the NADH-bound P2Y14–G_i_ complex, revealing the NADH molecular recognition mechanism. This suggests that NADH functions as a paracrine signal through P2Y14, playing crucial roles in immune and inflammatory responses. In conclusion, our findings provide important insights into receptor–ligand interactions and downstream transducer coupling mechanisms in P2Y14, offering a foundation for structure-based drug design.

## Supplementary information

Supplementary Information

## Data Availability

The cryo-EM density maps have been deposited in the Electron Microscopy Data Bank (EMDB) and Protein Data Bank (PDB) under accession numbers EMD-61052, EMD-63224 (composite) and 9J0B for UDPG–P2Y14–G_i_ complex; EMD-61055 and 9J0I for UDPGA–P2Y14–G_i_ complex; EMD-61054, EMD-63223 (composite) and 9J0F for NADH–P2Y14–G_i_ complex.
