# Supplementary material for: Structural insights into ligand recognition and activation of human purinergic receptor P2Y14
Source: Cell Discov. 2025 May 13;11:48. doi: 10.1038/s41421-025-00799-9 (PMC12075594; doi:10.1038/s41421-025-00799-9)
Supplement: Supplementary file 1 — Supplementary Information [file 41421_2025_799_MOESM1_ESM.pdf]

Supplementary Information

Structural insights into ligand recognition and activation of human purinergic receptor P2Y14

Quanchang Gu[1,2,3#], Zhenyu Lv[2#], Tianxin Wang[4,5#], Wenqin Tang[2,3#], Xuzhen Guo[1], Xiangling Huang[2], Fahui Li[2,3]*, Jiangyun Wang[2,3,4]*

[1]CAS Key Laboratory of Quantitative Engineering Biology, Institute of Synthetic Biology, Shenzhen Institute of Advanced Technology, Chinese Academy of Sciences, Shenzhen 518055, China

[2]Key Laboratory of Biomacromolecules (CAS), CAS Center for Excellence in Biomacromolecules, Institute of Biophysics, Chinese Academy of Sciences, Beijing 100101, China

[3]School of Life Sciences, University of Chinese Academy of Sciences, Beijing 100049, China.

[4]iHuman Institute, ShanghaiTech University, Shanghai 201210, China

[5]School of Life Science and Technology, ShanghaiTech University, Shanghai 201210, China

*Corresponding author. Email: jwang@ibp.ac.cn, lifahui@moon.ibp.ac.cn

# These authors contributed equally: Quanchang Gu, Zhenyu Lv, Tianxin Wang, Wenqin Tang

Materials and Methods

Constructs

For the cryo-EM structure determination of the P2Y14-G_i complex, the human wild-type *P2Y14* gene (amino acids 2-338) was cloned into a modified pFastBac1 vector with N-terminal HA signal peptide, FLAG tag, 10×His tag and a tobacco etch virus (TEV) protease site. To enhance receptor expression, a BRIL tag was fused to the N-terminal of P2Y14. Dominant-negative G_{i1} (S47N, G203A, E245A, and A326S) was generated based on wild-type G_{i1} following a previous report.[1] Human wild-type $G\beta_1$ and $G\gamma_2$ were subcloned into the pFastbac dual vector. Additionally, scFv16 was cloned into pFastBac1 with the C-terminal 6xHis tag and the N-terminal GP67 signal peptide. The $G_{q/i}$ chimera was constructed by replacing the α-helix 5 of G_q with that of G_{i1}, and subsequently subcloned into the pCDNA3.1 vector.

Purification of scFv16

The expression and purification of scFv16 were performed similarly to previously described methods. The scFv16 bacmid was transfected into *Spodoptera frugiperda sf*9 insect cells to generate recombinant baculovirus using Bac-to-Bac system (Invitrogen). *Trichuplusia ni* Hi5 insect cells were then infected with the baculovirus to express scFv16. The supernatant was collected, adjusted to pH using 1 M Tris (pH 8.0), and loaded onto Ni-FF resin. The column was washed with 15 column volumes (CVs) of Washing Buffer I (20 mM HEPES pH 7.5, 100 mM NaCl, 30 mM imidazole) followed by 15 CVs of Washing Buffer II (20 mM HEPES pH 7.5, 100 mM NaCl, 50 mM imidazole), and then eluted with 6 CVs of Elution Buffer (20 mM HEPES pH 7.5, 100 mM NaCl, 300 mM imidazole). The scFv16 protein was concentrated and further purified using a Superdex 75 Increase 10/300 GL column (Cytiva) in running buffer

(20 mM HEPES pH 7.5, 100 mM NaCl). The scFv16 peak fractions were collected, concentrated, and flash frozen for subsequent use.

Expression and purification of P2Y14-G_{i1} protein complexes

The P2Y14 receptor, along with $G\alpha_{i1}$ and $G\beta_1\gamma_2$, was co-expressed in *Sf9* cells using the Bac-to-Bac baculovirus expression system. The *Sf9* cells were cultured in ESF 921 medium (Expression Systems) to a density of 2.5×10^6 cells/mL, and infected with three separate virus preparations in a 1:1:1 ratio for P2Y14, $G\alpha_{i1}$, and $G\beta_1\gamma_2$. After 48 hours, cell pellets were harvested and stored at -80°C until use. The cell biomass (0.5 L) was resuspended and lysed in a buffer containing 10 mM HEPES pH 7.5, 10 mM $MgCl_2$, 20 mM KCl, and EDTA-free protease inhibitor cocktail (TargetMol). The lysate was supplemented with 25 μM UDPG, 50 μM UDPGA, or 100 μM NADH. To initiate complex formation, scFv16 was added to the P2Y14-$G\alpha_{i1}$ complexes at a final concentration of 10 μg/mL, and the mixture was incubated at 4 °C for 3 hours. The mixture was then centrifuged at 35,000 rpm for 40 minutes. The resulting pellet was solubilized in a buffer containing 50 mM HEPES pH 7.5, 100 mM NaCl, 0.6 % (w/v) lauryl maltose neopentyl glycol (LMNG, Anatrace), and 0.12 % (w/v) cholesterol hemisuccinate (CHS, Sigma-Aldrich), along with 25 μM UDPG, 50 μM UDPGA, or 100 μM NADH. After incubation at 4°C for 3 hours, the mixture was centrifuged again at 35,000 rpm for 40 minutes. The supernatant was collected and incubated overnight with 0.6 mL TALON IMAC resin (Clontech) in the presence of 20 mM imidazole at 4 °C. The resin was pelleted by centrifugation at 800 g for 5 minutes and loaded onto a gravity column. It was washed with 15 column volumes (CVs) of Washing Buffer I (25

mM HEPES pH 7.5, 100 mM NaCl, 10 % (v/v) glycerol, 0.1 % (w/v) LMNG, 0.02 % (w/v) CHS, 30 mM imidazole, and the corresponding ligand), followed by 15 CVs of Washing Buffer II (25 mM HEPES pH 7.5, 100 mM NaCl, 10 % (v/v) glycerol, 0.03 % (w/v) LMNG, 0.006 % (w/v) CHS, 50 mM imidazole, and the corresponding ligand). The complexes were then eluted three times with 3 CVs of Elution Buffer (25 mM HEPES pH 7.5, 100 mM NaCl, 10 % (v/v) glycerol, 0.01 % (w/v) LMNG, 0.002 % (w/v) CHS, 250 mM imidazole, and the corresponding ligand). The P2Y14-G_i complexes were concentrated and further purified on a Superdex 200 Increase 10/300 GL column (Cytiva) pre-equilibrated with running buffer (20 mM HEPES pH 7.5, 100 mM NaCl, 0.00075 % (w/v) LMNG, 0.00025 % (w/v) CHS, 0.00025 % (w/v) GDN, 100 μM TCEP, and the corresponding ligand). Fractions with a component ratio close to 1:1:1:1 were collected and concentrated to approximately 2 mg/mL.

Cryo-EM grid preparation and data collection

For the P2Y14-G_i complex, 3 μL of the sample was applied to glow-discharged holey gold grid (UltrAuFoil 300 mesh, R1.2/1.3), and vitrified in liquid ethane using a Vitrobot Mark IV (Thermo Fisher Scientific). The grid was blotted for 6 seconds with a blot force of -10 under conditions of 100 % humidity and 4 °C. Cryo-EM movie stacks were collected on a Titan Krios microscope operated at 300 kV, equipped with a Gatan K3 summit direct electron camera and a Gatan energy filter (slit set to 20 eV). All datasets were recorded at a nominal magnification of 105,000 in super-resolution mode, yielding a pixel size of 0.416 Å. The total electron dose is ~60 $e^-/Å^2$, divided into 40 frames. Each exposure lasted 2.77 seconds, with the dose rate of 15 $e^-/Å^2/s$. Defocus

ranges and dataset sizes were detailed in Supplementary Table S1.

Image processing and map construction

The movies were firstly imported into RELION 4.0[2] and beam-induced motion correction was performed with *MotionCor2*[3], binning 2 to physical pixel size of 0.832 Å. The corrected images were then imported into cryosparc v4[4], Patch CTF estimated was performed and images with CTF>5.0 Å was excluded from further processing. For then UDPG-P2Y14-G_i-scFv16 dataset, the remaining pictures were subjected to blob picking with a size range from 120 Å to 160 Å. The extracted particles were classified in 2D. Obvious GPCR-G protein complex 2D classes were selected for 3D Ab-initio reconstruction and heterogeneous refinement into 5 volume classes. Particles from then best class were selected for a second round of 3D classification. The best volume was subjected to non-uniform Refinement, and a 2D template was created to re-pick particles using the template picker. Particles were extracted with 300 pixels and rescaled to 150 pixels for the first round of 3D ab initio reconstruction and heterogeneous refinement. The previously used 3D template volume was low-pass filtered and used as one of five classes in heterogeneous refinement. The classification performed well, selecting 1,030,081 particles, which were re-extracted with a box size of 320 pixels. These particle sets underwent non-uniform refinement, achieving a global resolution of 2.58 Å, although the receptor region was poorly resolved. Further 3D classification was performed in CryoSPARC v4, with a mask focused on the receptor, created using ChimeraX. Further 3D classification was performed in CryoSPARC v4, with a mask focused on the receptor, created using ChimeraX[5]. In the first round of 3D classification,

10 volume classes were generated; volumes with incomplete TMD helices were discarded, and the remaining particles were subjected to a second round of 3D classification, generating three classes that underwent non-uniform refinement. The best volume was chosen for local refinement, focusing on the complex or receptor. For the UDPGA-P2Y14-G_i-scFv16 dataset, particle picking was performed using the 2D templates created from the UDPG-P2Y14-G_i-scFv16 volume. After two rounds of ab initio reconstruction and heterogeneous refinement, 1,019,044 particles were retained and subjected to 3D classification focused on the receptor region. Particles from one of the ten classes were selected for non-uniform refinement and local refinement to obtain the final volume. For the NADH-P2Y14-G_{i1} dataset, preliminary 2D templates were created ab initio, similar to the UDPG-P2Y14-G_{i1}-scFv16 dataset. Particles were first picked based on theoretical size, classified in 2D, and obvious 2D classes corresponding to the complex were selected. Ab initio reconstruction and heterogeneous refinement were used for initial 3D classification, followed by re-extraction of particles from the best volume and non-uniform refinement. The resulting volume was used to create 2D templates, which were used for template picking. After two rounds of ab initio reconstruction and heterogeneous refinement, best volumes and corresponding particles underwent non-uniform and local refinement to generate the final volume.

Model building

For all complexes, the coordinates of the P2Y14 AlphaFold2[6]-predicted model and the G_i heterotrimer-scFv16 complex from PDB 8IRT were used as initial models for refinement. These initial models were rigidly fitted into the electron microscopy density

map using ChimeraX. The coordinates were then manually refined using COOT[7] and automatically refined with Phenix.real_space_refine[8] or ISOLDE[9]. We used the composite map generated by Phenix.Combine_Focused_Maps to refine the models of the UDPG- and NADH-bound P2Y14 complexes. Model validation was performed using MolProbity[10] in Phenix.real_space_refine. The regions of poor density or unmodeled side chains in the P2Y14 receptor for each ligand-bound model include: UDPG-bound P2Y14 model: N-terminal (1-15), I37, L38, L84, W87, T129, F131, I132, Q133, V135, S136, V154, S163, V164, R165, E166, Q169, E174, E178, K182, K211, L218, R222, K229, R233, E262, H264, S266, C267, Q268, Q300, and the C-terminal loop (311-338); UDPGA-bound P2Y14 model: N-terminal loop (1-14), I24, M31, F48, K54, L84, Q88, R95, K122, L127, T129, S130, F131, I132, V135, S136, Y137, L140, I144, L148, L150, L151, V154, N161, V164, R165, E166, V167, T168, Q169, I170, E174, E178, R181, K182, L200, K215, L218, K219, S220, S221, R222, K229, K230, R233, E262, Q268, K270, F297, C307, K308, K309, and the C-terminal loop (312-338); NADH-bound P2Y14 model: N-terminal loop (1-16), N17, L19, Q23, L38, K54, R95, L100, K125, T129, S130, Q133, V135, R165, E166, E174, K182, K211, R222, E262, Q300, E304, and the C-terminal loop (312-338).

MD setting

All simulation models were constructed based on the ligand-bound state of the receptor, with any missing heavy atoms repaired in COOT. MD settings were configured using the CHARMM-GUI Membrane Builder[11] workflow. In this pipeline, models were titrated to pH 7.0 using PropKa[12], and a disulfide bond between residues

C172 and C94 was fixed. Models were reoriented perpendicular to the membrane plane using OPM 2.0[13] and embedded into a 100-palmitoyl-oleoyl-phosphatidylcholine (POPC) bilayer. TIP3P water models and 0.15 M Na^+/Cl^- ion pairs were added on both sides of the lipid bilayer to neutralize the charge at a height of 22.5 Å. The CHARMM36 force field[14] was employed for protein molecules, lipids, water, and salt ions. Ligand parameters were generated using the CHARMM General Force Field (CGenFF)[15], with a net charge of -2. MD simulations were conducted using GROMACS 2024.1[16]. For each system, minimization and equilibration stages followed the CHARMM-GUI suggested scheme, with a reduced position restraint force applied for a total of 1.375 ns. Production runs were conducted in the NPT ensemble with Parrinello-Rahman semi-isotropic pressure coupling at 1 bar and V-rescale temperature coupling at 310.15 K for 500 ns. The integration timestep was 2 fs, with covalent hydrogen constraints applied using the LINCS[17] method. The cutoffs for van der Waals and Coulomb interactions were set to 12 Å, and long-range interactions were calculated using the PME method[18].

Receptor cell surface expression assay

To assess the cell surface expression of the P2Y14 wild type and its mutants, HEK293T cells (ATCC CRL-3216) were transfected with the respective plasmids using PEI 25K (Polysciences 23966). The plasmid amounts were consistent with those used in the calcium-induced luciferase accumulation assay. After 24 hours, the cells were harvested, washed twice with PBS, and incubated with anti-DYKDDDK-APC (BioLegend, 637308) at 4°C for one hour. Following incubation, the cells were washed twice with PBS and analyzed using a BD Calibur cytometer.

Calcium-induced luciferase accumulation assay

HEK293T cells were cultured in a 24-well plate at a density of 1×10^5 cells per well in an incubator. After overnight growth, the cells were transfected with pGL4.30 [luc2P/NFAT-RE/Hygro], $G_{q/i}$ chimera, and either wild-type or mutant P2Y14 using PEI 25K (Polysciences 23966) as the transfection reagent for 24 hours. The cells were then treated with varying concentrations of UDPG, UDPGA, NADH, and NAD^+ for 12 hours. Luciferase activity was measured using a luciferase reporter assay kit (Promega) and normalized to the total protein content of the cell lysates. Data were analyzed using the log(agonist) vs. normalized response (variable slope) function in GraphPad Prism 10. Experiments were conducted in triplicate, and results are presented as mean ± s.e.m.

REFERENCES

1 Draper-Joyce, C. J. et al. *Nature*, **558**, 559-563 (2018).
2 Zivanov, J. et al. *eLife*, **7**, e42166 (2018).
3 Zheng, S. Q. et al. *Nat Methods*, **14**, 331-332 (2017).
4 Punjani, A., Rubinstein, J. L., Fleet, D. J. & Brubaker, M. A. *Nat Methods*, **14**, 290-296 (2017).
5 Meng, E. C. et al. *Protein Sci*, **32**, e4792 (2023).
6 Varadi, M. et al. *Nucleic Acids Res*, **50**, D439-D444 (2022).
7 Emsley, P., Lohkamp, B., Scott, W. G. & Cowtan, K. *Acta Crystallogr D Biol Crystallogr*, **66**, 486-501 (2010).
8 Afonine, P. V. et al. *Acta Crystallogr D Struct Biol*, **74**, 531-544 (2018).
9 Croll, T. I. *Acta Crystallogr D Struct Biol*, **74**, 519-530 (2018).
10 Williams, C. J. et al. *Protein Sci*, **27**, 293-315 (2018).
11 Lee, J. et al. *J Chem Theory Comput*, **12**, 405-413 (2016).
12 Olsson, M. H. M., Søndergaard, C. R., Rostkowski, M. & Jensen, J. H. *J Chem Theory Comput*, **7**, 525-537 (2011).
13 Lomize, M. A., Pogozheva, I. D., Joo, H., Mosberg, H. I. & Lomize, A. L. *Nucleic Acids Res*, **40**, D370-D376 (2012).
14 Huang, J. et al. *Nat Methods*, **14**, 71-73 (2017).
15 Vanommeslaeghe, K. & MacKerell, A. D., Jr. *J Chem Inf Model*, **52**, 3144-3154 (2012).
16 Abraham, M. J. et al. *SoftwareX*, **1-2**, 19-25 (2015).

17 Hess, B., Bekker, H., Berendsen, H. J. C. & Fraaije, J. G. E. M. *J Chem Theory Comput*, **18**, 1463-1472 (1997).
18 Essmann, U. et al. *J Chem Phys*, **103**, 8577-8593 (1995).

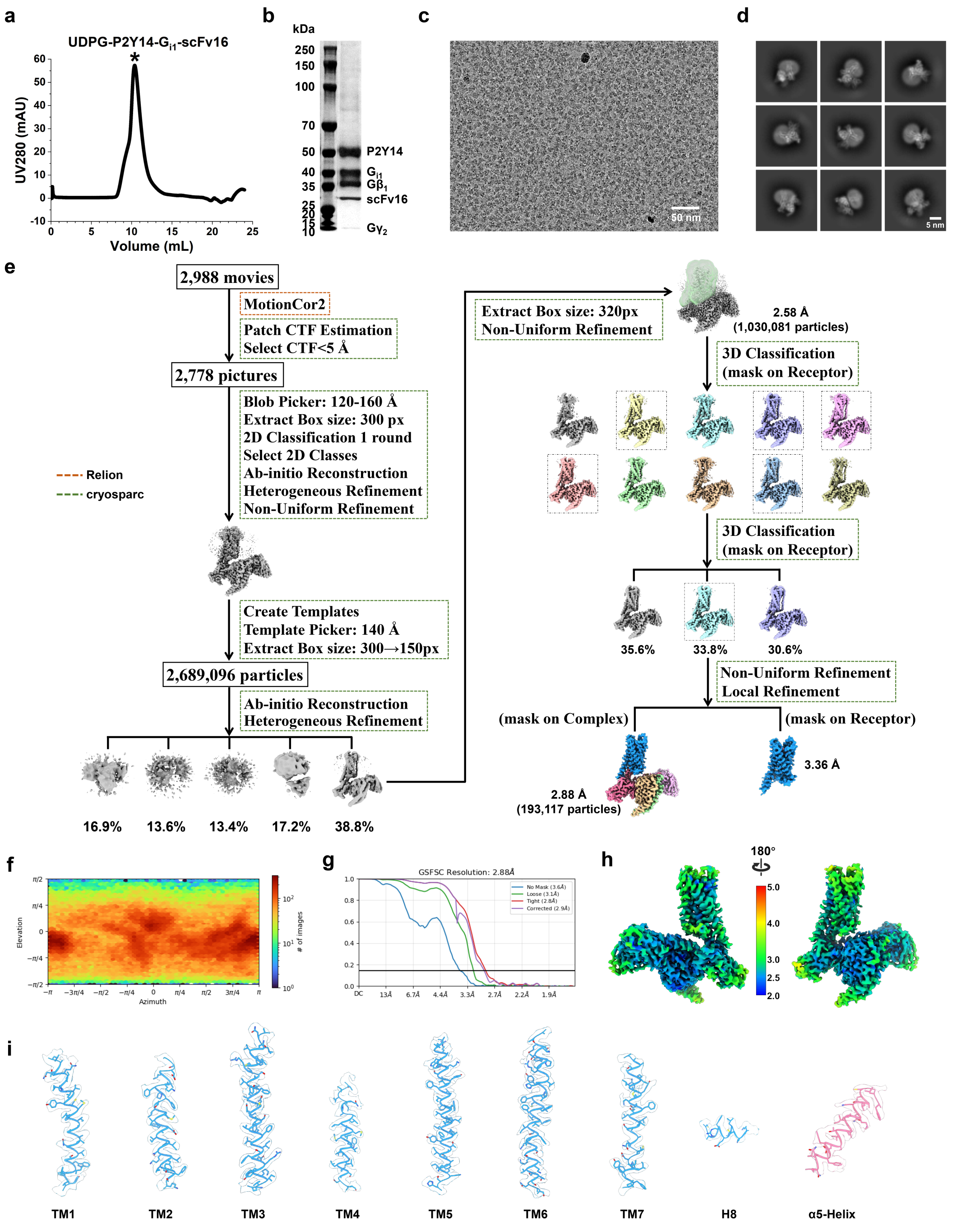

Supplementary Fig. S1 Sample preparation and cryo-EM data processing of the UDPG-bound P2Y14-G$_i$ complex. a Representative size-exclusion chromatography elution profile of the purified UDPG-bound P2Y14-G$_i$ complex. **b** SDS-PAGE and Coomassie blue staining analysis of the size-exclusion chromatography peak for the UDPG-bound P2Y14-G$_i$ complex. **c** Representative cryo-EM image of the purified

UDPG-bound P2Y14-G_i complex. **d** Two-dimensional (2D) class averages of the UDPG-bound P2Y14-G_i complex. **e** Flowchart of three-dimensional (3D) reconstitution of the UDPG-bound P2Y14-G_i complex. **f** Angular distribution of particles used for final reconstruction. **g** Fourier shell correlation (FSC) curves for the final 3D density map. **h** 3D density map colored according to local resolution (Å) of the UDPG-bound P2Y14-G_i complex. **i** Model-map fitting quality of the transmembrane α-helices (TM1-7) and helix8 of P2Y14 and the α5-helix of G_i in the UDPG-bound complex.

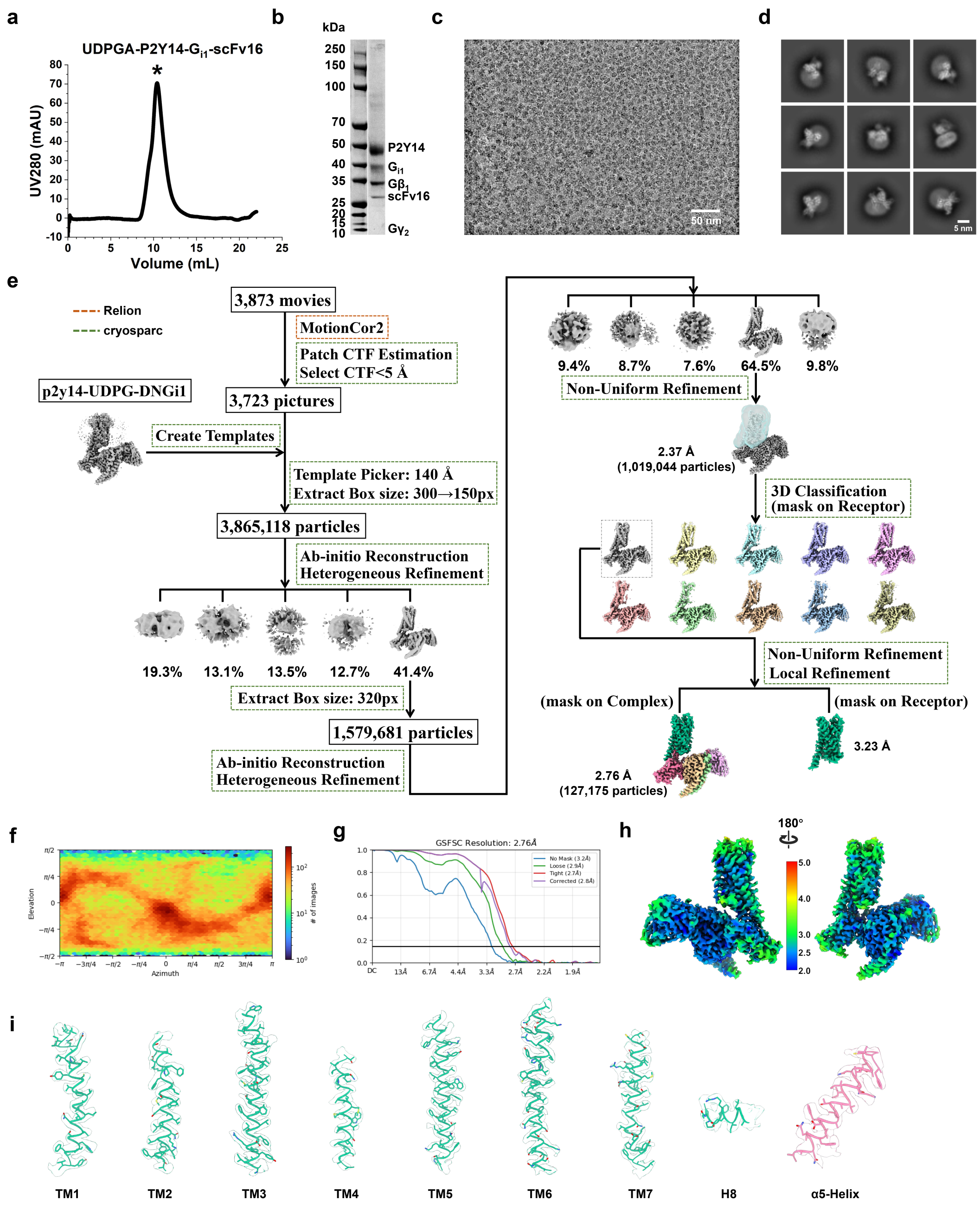

Supplementary Fig. S2 Sample preparation and cryo-EM data processing of the UDPGA-bound P2Y14-G_i complex. a Representative size-exclusion chromatography elution profile of the purified UDPGA-bound P2Y14-G_i complex. **b** SDS-PAGE and Coomassie blue staining analysis of the size-exclusion chromatography peak of the UDPGA-bound P2Y14-G_i complex. **c** Representative cryo-EM image of the purified UDPGA-bound P2Y14-G_i complex. **d** Two-dimensional (2D) class averages of the

UDPGA-bound P2Y14-G_i complex. **e** Flowchart of three-dimensional (3D) reconstitution of the UDPGA-bound P2Y14-G_i complex. **f** Angular distribution of the particles used for final complex reconstruction. **g** Fourier shell correlation (FSC) curves for the final 3D density map. **h** 3D density map colored according to local resolution (Å) of the UDPGA-bound P2Y14-G_i complex. **i** Model-map fitting quality of the transmembrane α-helices (TM1-7) and helix8 of P2Y14 and the α5-helix of G_i in the UDPGA-bound complex.

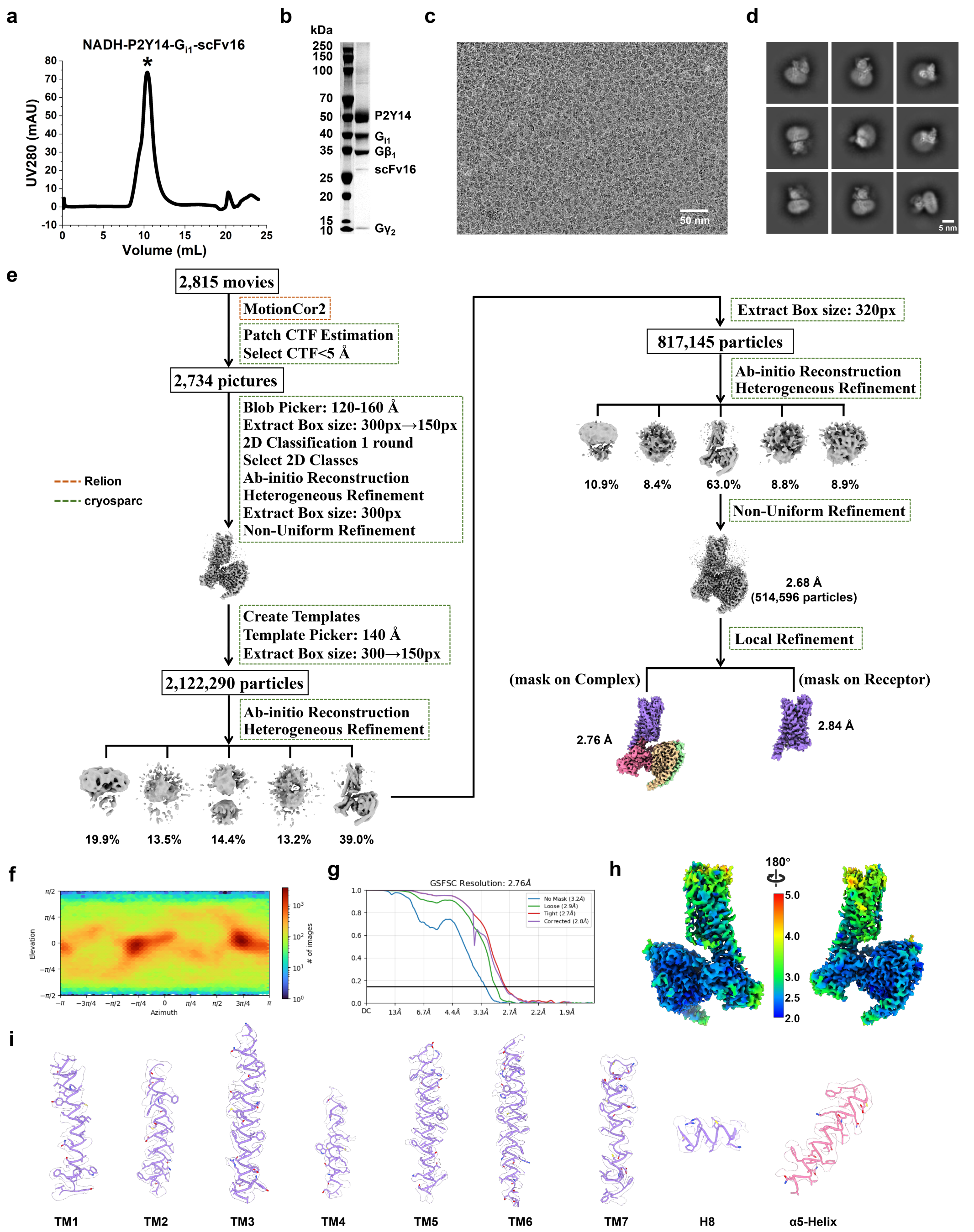

Supplementary Fig. S3 Sample preparation and cryo-EM data processing of the NADH-bound P2Y14-G$_i$ complex. a Representative size-exclusion chromatography elution profile of the purified NADH-bound P2Y14-G$_i$ complex. **b** SDS-PAGE and Coomassie blue staining analysis of the size-exclusion chromatography peak of the NADH-bound P2Y14-G$_i$ complex. **c** Representative cryo-EM image of the purified

NADH-bound P2Y14-G_i complex. **d** Two-dimensional (2D) class averages of the NADH-bound P2Y14-G_i complex. **e** Flowchart of three-dimensional (3D) reconstitution of the NADH-bound P2Y14-G_i complex. **f** Angular distribution of the particles used for final reconstruction. **g** Fourier shell correlation (FSC) curves for the final 3D density map. **h** 3D density map colored according to local resolution (Å) of the NADH-bound P2Y14-G_i complex. **i** Model-map fitting quality of the transmembrane α-helices (TM1-7) and helix8 of P2Y14 and the α5-helix of G_i in the NADH-bound complex.

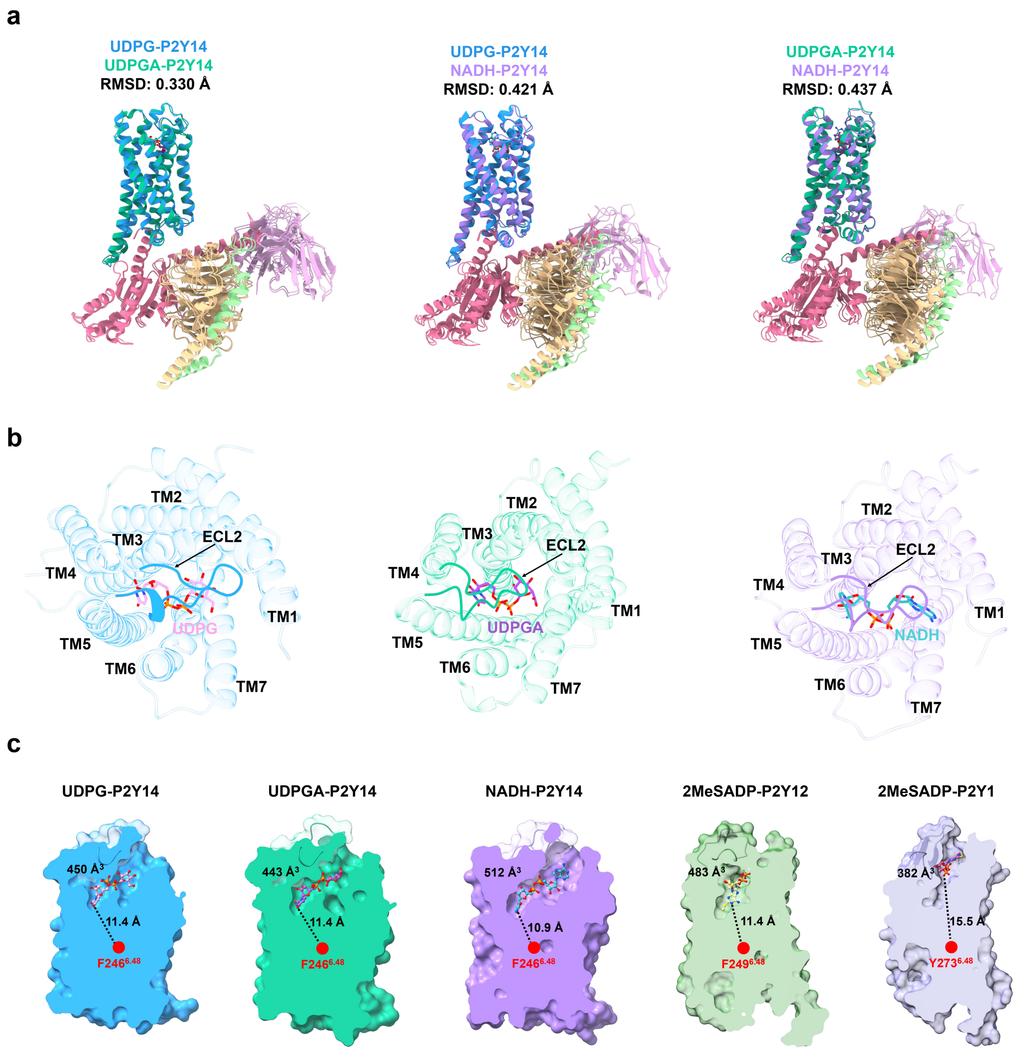

Supplementary Fig. S4 Overall structures of P2Y14 complexes. a Structural comparison of UDPG-, UDPGA- and NADH-bound P2Y14 complexes, with root mean square deviation (RMSD) values calculated for pairwise comparison. **b** Extracellular views of the UDPG-, UDPGA-, and NADH-bound P2Y14 structures, showing ECL2 anchoring the agonists in the orthosteric cavity. **c** Cut-away view of the ligand-binding pocket in the UDPG-, UDPG-, NADH-bound P2Y14-G_i complexes, as well as the 2MeSADP-bound P2Y12-G_i (PDB code: 7XXI) and P2Y1-G_{11} (PDB code: 7XXH) complexes. The distances between ligands and the toggle switch 6.48 range from 10.9 to 15.5 Å, with ligand-binding pocket volumes ranging from 382 to 512 Å³.

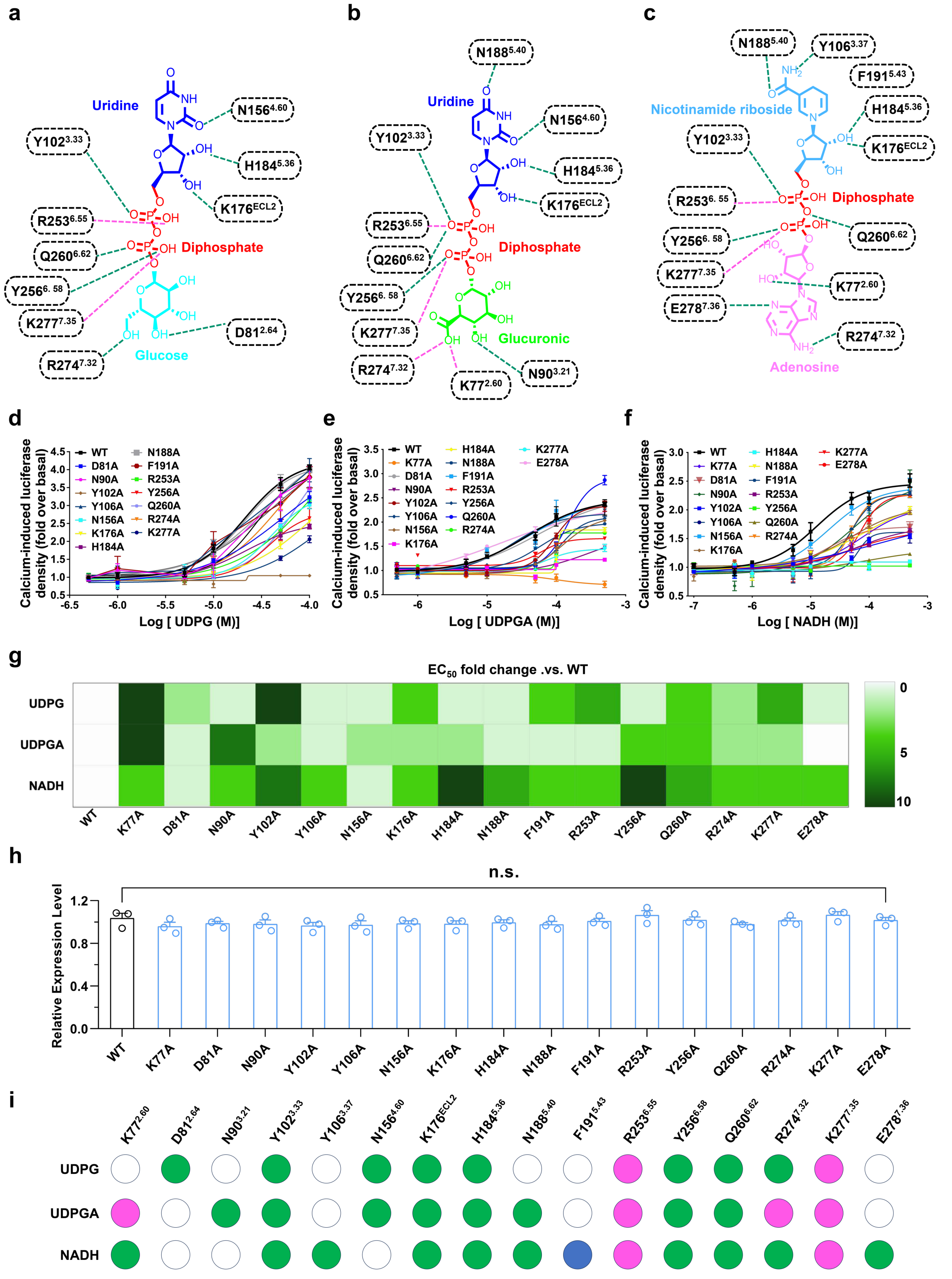

Supplementary Fig. S5 Agonists recognition mechanism of P2Y14. a-c 2D representation of interactions between P2Y14 and UDPG (**a**), UDPGA (**b**), and NADH (**c**). Hydrogen bonding interactions are indicated with green dashed lines, and ionic interactions are indicated with pink dashed lines. **d-f** Mutational effects of P2Y14's orthosteric pocket on calcium response activities induced by UDPG (**d**), UDPGA (**e**),

and NADH (**f**). Data are shown as the mean ± s.e.m. from three independent measurements. **g** Heatmap representation of the effect of mutations in the ligand binding pocket on potency (EC_{50}) of P2Y14 towards UDPG, UDPGA, and NADH. The changes of EC_{50} (mutant vs. wild type) were indicated by the green color scale. The heatmap is generated according to the data shown in Fig. S5d-f and Table S2. **h** Flow cytometry assays showing similar expression levels of the P2Y14 wild type and its mutants in the ligand binding pocket. Values are the mean ± s.e.m. from three independent experiments. n.s., no significant difference. All data were analyzed by two-sided one-way ANOVA with Tukey test. **i** Interaction differences in the orthosteric pocket between UDPG-, UDPGA-, and NADH-bound P2Y14. Residues involved in hydrogen bonding, ionic, and hydrophobic interactions are indicated by green, pink, and blue dots, respectively, while residues without interactions are shown as white dots.

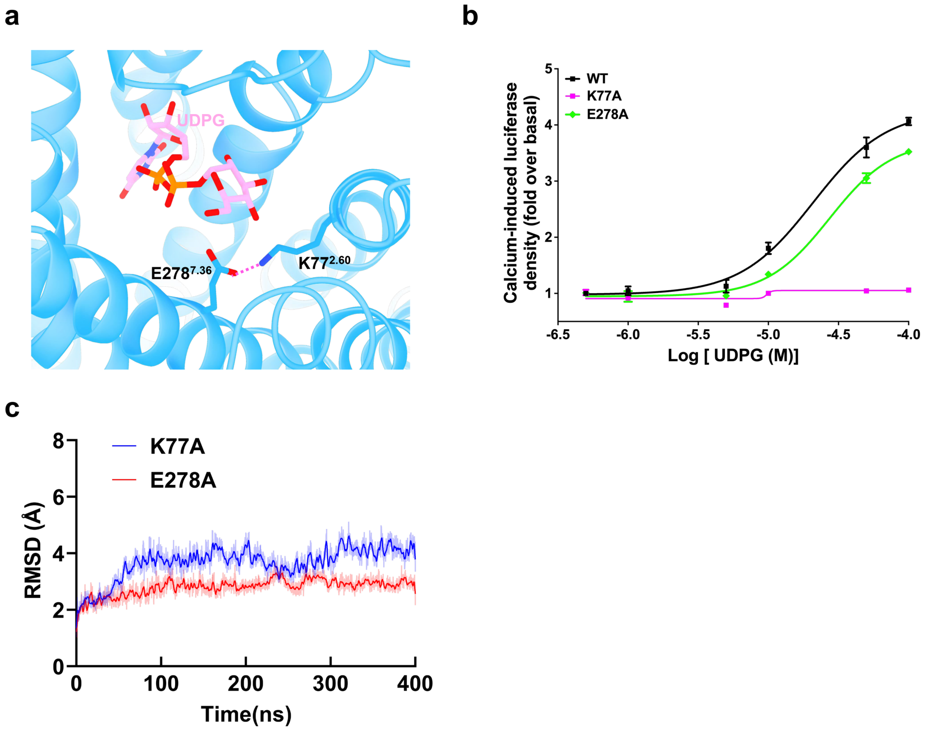

Supplementary Fig. S6 The role of residue K77$^{2.60}$ in UDPG-bound P2Y14 structure. a Interaction between K77$^{2.60}$ and E278$^{7.36}$ in UDPG-bound P2Y14 structure. The salt bridge between K77$^{2.60}$ and E278$^{7.36}$ is crucial for maintaining the architecture of the extracellular cavity. Ionic interaction is indicated with pink dashed line. **b** Effects of mutations at the P2Y14 on response to UDPG. Data are shown as the mean ± s.e.m. from three independent measurements. **c** MD simulations of UDPG-bound P2Y14 K77A and E278A mutants. Average RMSD values of backbones in UDPG-bound P2Y14 K77A and E278A mutants during 400 ns MD simulation.

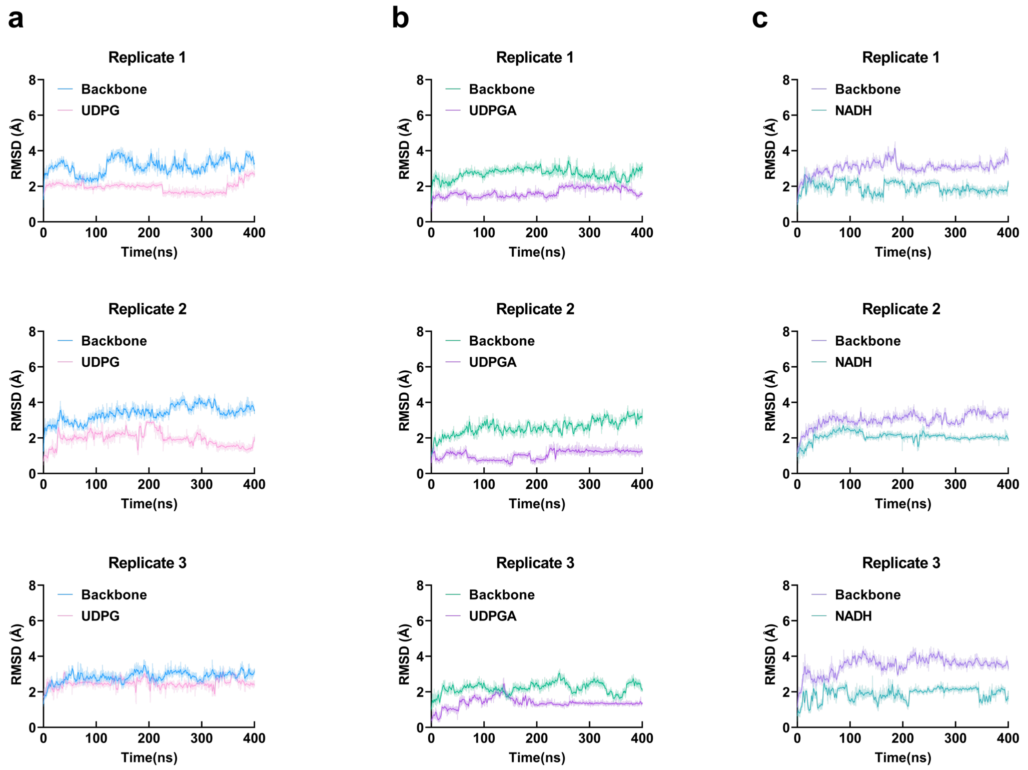

Supplementary Fig. S7 MD simulations of UDPG-, UDPGA- and NADH-bound P2Y14-G$_i$ complexes. a-c Average RMSD values of ligands and backbones in UDPG (**a**)-, UDPGA (**b**)-, and NADH (**c**)-bound P2Y14 during triplicate 400 ns MD simulations.

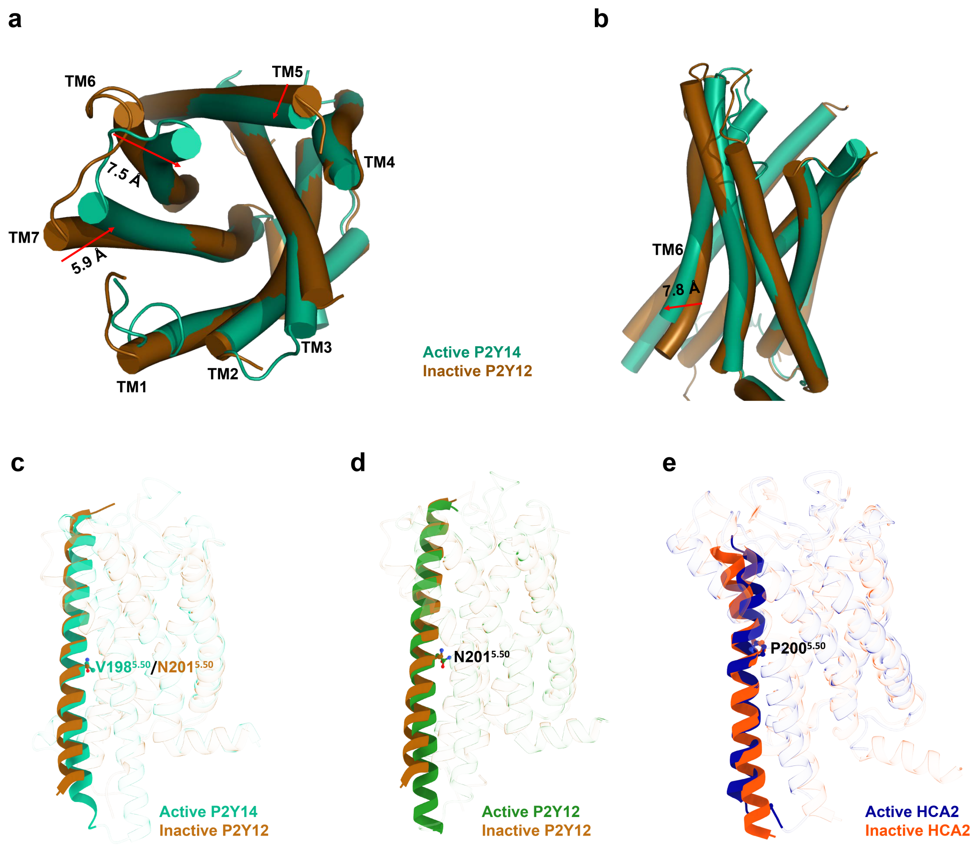

Supplementary Fig. S8 Activation of P2Y14. a, b Extracellular (**a**) and side (**b**) views comparing the UDPGA-bound P2Y14 and the AZD1283-bound inactive P2Y12 (PDB: 4NTJ) structures. Conformational changes are indicated by red arrows. **c-e** Side views of TM5 alignment: active P2Y14 with inactive P2Y12 (**c**), active P2Y12 (PDB: 7XXI) with inactive P2Y12 (**d**), and active HCA2 (PDB: 7XK2) with inactive HCA2 (PDB: 7ZLY) (**e**).

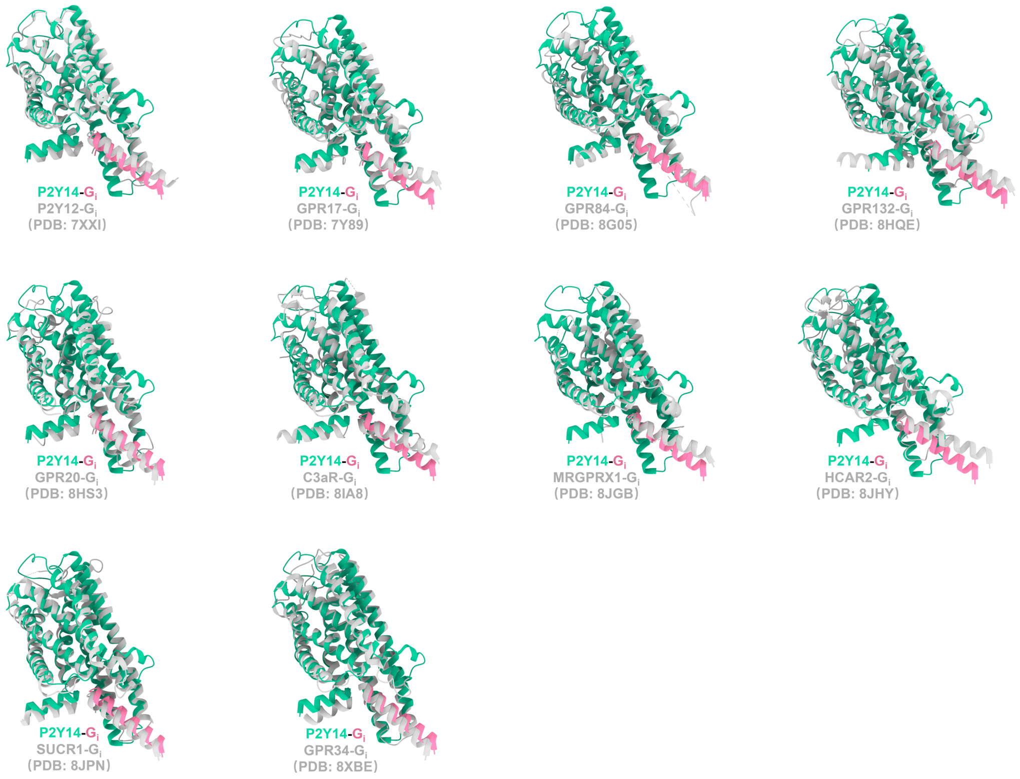

Supplementary Fig. S9 Structural comparison of GPCR-G_i complexes. The UDPGA-bound P2Y14 structure is used as the reference for comparison.

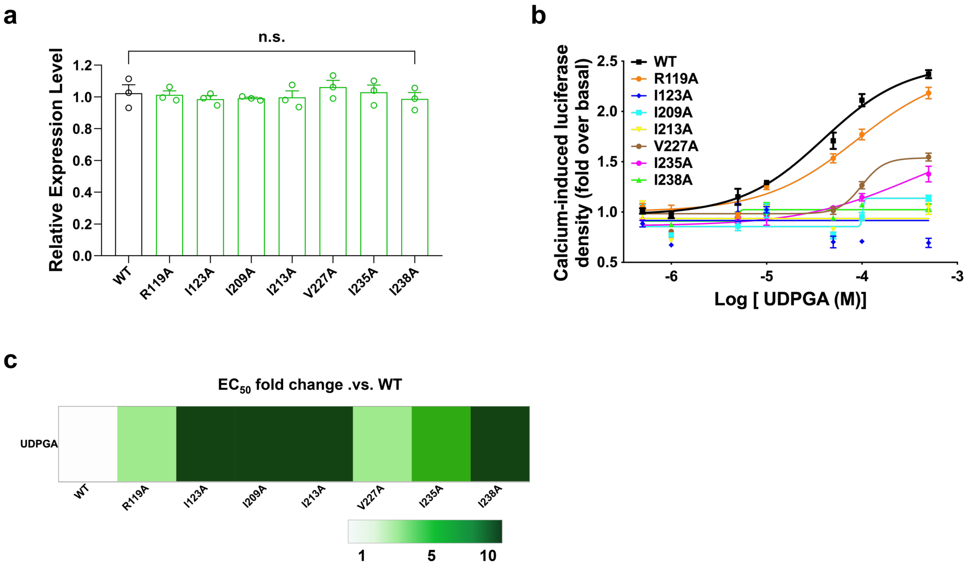

Supplementary Fig. S10 G_i-coupling mechanism of P2Y14. a Flow cytometry assays showing similar expression levels of the P2Y14 wild type and its mutants in G_i interface. Values are the mean ± s.e.m. from three independent experiments. n.s., no significant difference. All data were analyzed by two-sided one-way ANOVA with Tukey test. **b** Mutational effect of P2Y14's G_i coupling interface on UDPGA induced calcium activities. Data are shown as the mean ± s.e.m. from three independent measurements. **c** Heatmap representation of the effect of mutations in receptor-G_i interface on potency (EC_{50}) of P2Y14 towards UDPGA. The changes of EC_{50} (mutant vs. wild type) were indicated by the green color scale. The heatmap is generated according to the data shown in Fig. S5e and Table S2.

Supplementary Table S1 Cryo-EM data collection, refinement and validation statistics.

	UDPG-P2Y14	UDPGA-P2Y14-	NADH-P2Y14
	(EMD-61052)<br>(PDB: 9J0B)	(EMD-61055)<br>(PDB: 9J0I)	(EMD-61054)<br>(PDB: 9J0F)
**Data collection and processing**			
Magnification	105,000	105,000	105,000
Voltage (kV)	300	300	300
Electron exposure (e-/Å2)	60	60	60
Defocus range (um)	-0.8 to -1.6	-0.9 to -1.3	-0.8 to -1.4
Pixel size (Å)	0.832	0.832	0.832
Symmetry imposed	C1	C1	C1
Initial particle projections (no.)	2,689,096	3,865,118	2,122,290
Final particle projections (no.)	193,117	127,175	173,360
Map resolution (Å)	2.88	2.76	2.76
Map resolution range (Å)	1.79-4.43	1.80-4.49	1.80-6.31
FSC threshold	0.143	0.143	0.143
**Model Refinement**			
Refinement package	phenix 1.20.1, isolde 1.8	phenix 1.20.1, isolde 1.8	phenix 1.20.1, isolde 1.8
Real or reciprocal space	Real	Real	Real
Model-Map CC (mask)	0.81	0.86	0.78
Model resolution (Å)	3.2	2.9	3.0
FSC threshold	0.5	0.5	0.5
B factor (Å2, min/max/mean value)			
Protein residues	0.00/93.74/33.76	0.00/110.86/29.28	0.00/88.06/27.54
Ligands	38.30/54.01/45.72	57.34/78.18/66.94	22.47/45.98/36.54
Model composition			
Non-hydrogen atoms	8599	8529	6855
Protein residues	1141	1142	905
R.m.s. deviations			
Bond lengths (Å)	0.004	0.004	0.004
Bond angles (°)	0.931	0.936	0.979
**Validation**			
MolProbity score	1.61	1.44	1.78
Clashscore	8.80	6.53	9.89
Rotamer outliers (%)	0.12	0.00	0.15
Ramachandran plot			
Favored (%)	97.24	97.60	96.08
Allowed (%)	2.76	2.40	3.92
Disallowed (%)	0.00	0.00	0.00

Supplementary Table S2 EC_{50} and maximal response statistics of different ligands bound P2Y14 wild type and its mutants.

	UDPG		UDPGA		NADH	
**Receptors**	**EC_{50} (μM)**	**Maximal response**	**EC_{50} (μM)**	**Maximal response**	**EC_{50} (μM)**	**Maximal response**
WT	21.51±3.53	4.06±0.068	40.59±1.734	2.37±0.041	16.06±8.187	2.51±0.115
K77A	nd	nd	nd	nd	75.74±10.005**	1.94±0.014**
D81A	61.39±11.807*	3.23±0.157**	58.22±30.89	2.3±0.16	24.8±1.556	1.7±0.091**
N90A	28.17±3.927	3.77±0.186	292.8±48.761**	1.48±0.004***	76.04±7.657**	2.47±0.222
Y102A	nd	nd	94.88±0.688***	2.33±0.106	126.58±25.874*	1.54±0.043*
Y106A	36.72±10.16	3.98±0.187	48.61±9.962	2.27±0.155	59.98±4.023**	1.53±0.104**
N156A	42.42±4*	3.02±0.116**	99.89±18.115*	2.03±0.022**	18.12±4.592	2.38±0.171
K176A	102.93±13.426**	2.56±0.032***	100.38±0.227***	1.22±0.017***	50.76±2.787*	1.95±0.036**
H184A	27.65±11.71	2.41±0.068***	102.13±0.555***	1.84±0.046***	nd	nd
N188A	21.93±4.409	3.98±0.042	57.54±0.981**	1.96±0.025**	98.26±16.142*	2±0.063*
F191A	67.06±36.478	3.79±0.171	54.46±19.038*	2.37±0.046	49.97±0.441*	2.29±0.036
R253A	117.64±24.759*	3.16±0.072***	61.29±6.338*	1.66±0.015***	60.48±4.922**	1.63±0.047**
Y256A	37.51±8.614	2.65±0.276**	202.27±73.624	2.07±0.033**	nd	nd
Q260A	75.31±11.016**	3.47±0.053**	150.73±11.106***	2.87±0.097**	103.1±28.516*	1.23±0.015***
R274A	49.52±4.559**	3.87±0.021	103.43±1.338***	1.78±0.027***	49.36±2.97*	2.3±0.086
K277A	146.37±27.407*	2.06±0.097***	82.18±12.84*	1.47±0.075***	48.43±1.101*	1.56±0.064**
E278A	27.42±1.388	3.52±0.05*	18.95±6.901*	2.22±0.075	75.36±3.89**	2.26±0.021
R119A			87.38±13.937*	2.18±0.057		
I123A			nd	nd		
I209A			nd	nd		
I213A			nd	nd		
V227A			100.72±3.178***	1.55±0.039***		
I235A			266.2±80.106*	1.38±0.078***		
I238A			nd	nd		

Data are shown as mean ± s.e.m. from at least three independent experiments performed in triplicate. *$P < 0.05$, **$P < 0.01$ and ***$P<0.001$ (one-way ANOVA followed by Dunnett's posttest, compared with the response of wild type). The maximal response is reported as the maximum effect of P2Y14 wild type and its mutants. nd (not determined) refers to data where a robust concentration response curve could not be established within the concentration range tested.
